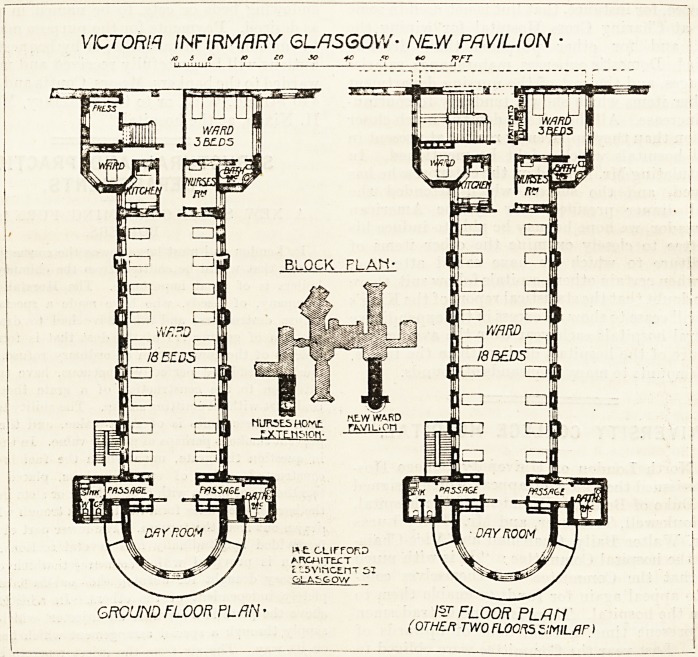# Additions to the Victoria Infirmary, Glasgow

**Published:** 1906-06-09

**Authors:** 


### ADDITIONS TO THE VICTORIA INFIRMARY, GLASGOW.

A four-story pavilion has lately been added to this Infirmary, and it was opened by Lady Blythewood in presence of the Lord Provost and a large assemblage of people. Lady Blythewood was presented with a gold key by Mr. Clifford, the key being the gift of the architect and the contractors.

The history of this Infirmary is not a long one; but it is a noteworthy one on account of its rapid development and the amount of good it has already done. The original building was opened in 1890, and it contained only 84 beds. A Nurses' Home was added in 1892, and next year accommodation was provided for 70 additional patients. A convalescent home for 24 patients was the next adjunct, and a very useful one it must have been. In 1902 the accommodation was again increased by 21 beds; but so rapidly did the work of the Infirmary increase that there was an average of 100 patients awaiting admission.

In 1903 it was therefore decided to increase the number of beds by about 80, and so raise the total accommodation to something like 260 beds. This conclusion was a wise one to arrive at.

The new block is four stories high. Its total length is about 140 feet, and its greatest width about 40 feet. On entering any one of the new floors there is on the left hand a ward for three beds, and on the right hand are the staircases and a storeroom of some kind. Passing through the door near the foot of the staircase we come to the door of the three-bedded ward, next to which is the nurses' room. On the right are the single-bedded ward and the ward-kitchen; the latter being exactly opposite the nurses' room. The main ward is entered between these. It is 74 feet long and 26 feet wide, and contains 18 beds, consequently each bed has a superficial space of 107 feet, and a wall space of 8 feet, 5 inches. It cannot be said that these measurements show too much space per bed. In our opinion the space is not sufficient for a modern hospital, but some latitude must be allowed, and we do not know what nature of cases is to be treated in these wards. Much depends on this. On the other hand, the ward has plenty of light, and every bed has a window on both sides. At the end of the ward are the sanitary annexes, and these are carefully cut off from the ward by cross-ventilated passages. Had we designed this part of the building we should have increased the length of these passages a little and cleared away the work which now projects into the ward and into the day-room; we should have replaced this work by glazed partitions, and thus brought the beautiful circular bays into full view from every part of the ward. Possibly the hot-air flues might have prevented this unless some other position could have been found for them. The circular bay, or day-room, is surrounded by a balcony, and all this part is not only useful but ornamental. Facing full south it is a most important adjunct to the ward.

Returning to the three-bedded ward it will be noticed that it has one large window and one smaller one, both on the same, in fact the only free, wall of the room. Hence, except when the door is open, there cannot be much cross-ventilation. Another faulty point about this three-bedded ward is that the room containing the bath, sink, and closet is not properly cut off from the ward, in fact, opens direct into it. The single-bedded ward is also without cross-ventilation other than by the door.

The three other floors are in all respects similar to the ground floor, and therefore need not be described. The total accommodation of these recent additions is for 76 beds, and there is space in the roof for 16 servants to sleep.

So far as we can see from the plans, there are no open fireplaces in any of the wards; and if this be so, the Plenum system is solely relied on for warming them. However successful this system may be as regards the distribution of warm air, it is not one that we should advise for sole use in any hospital. Open fireplaces should always be the chief sources of heat, and other appliances should be subsidiary thereto. It is stated that in the Victoria Infirmary the most approved methods of purifying the air have been introduced.

The cost of the additions was £30,000. The architect was Mr. Clifford, of Glasgow, and the contractors were Messrs. Emery and Son.

## Figures and Tables

**Figure f1:**